# Integration of clinical parameters and CT-based radiomics improves machine learning assisted subtyping of primary hyperaldosteronism

**DOI:** 10.3389/fendo.2023.1244342

**Published:** 2023-08-24

**Authors:** Nabeel Mansour, Andreas Mittermeier, Roman Walter, Balthasar Schachtner, Jan Rudolph, Bernd Erber, Vanessa F. Schmidt, Daniel Heinrich, Denise Bruedgam, Lea Tschaidse, Hanna Nowotny, Martin Bidlingmaier, Sonja L. Kunz, Christian Adolf, Jens Ricke, Martin Reincke, Nicole Reisch, Moritz Wildgruber, Michael Ingrisch

**Affiliations:** ^1^ Department of Radiology, LMU University Hospital, LMU Munich, Munich, Germany; ^2^ Department of Medicine IV, LMU University Hospital, LMU Munich, Munich, Germany

**Keywords:** machine learning, hyperaldosteronism, adrenal venous sampling, integrated diagnostics, venous interventions

## Abstract

**Objectives:**

The aim of this study was to investigate an integrated diagnostics approach for prediction of the source of aldosterone overproduction in primary hyperaldosteronism (PA).

**Methods:**

269 patients from the prospective German Conn Registry with PA were included in this study. After segmentation of adrenal glands in native CT images, radiomic features were calculated. The study population consisted of a training (n = 215) and a validation (n = 54) cohort. The k = 25 best radiomic features, selected using maximum-relevance minimum-redundancy (MRMR) feature selection, were used to train a baseline random forest model to predict the result of AVS from imaging alone. In a second step, clinical parameters were integrated. Model performance was assessed via area under the receiver operating characteristic curve (ROC AUC). Permutation feature importance was used to assess the predictive value of selected features.

**Results:**

Radiomics features alone allowed only for moderate discrimination of the location of aldosterone overproduction with a ROC AUC of 0.57 for unilateral left (UL), 0.61 for unilateral right (UR), and 0.50 for bilateral (BI) aldosterone overproduction (total 0.56, 95% CI: 0.45-0.65). Integration of clinical parameters into the model substantially improved ROC AUC values (0.61 UL, 0.68 UR, and 0.73 for BI, total 0.67, 95% CI: 0.57-0.77). According to permutation feature importance, lowest potassium value at baseline and saline infusion test (SIT) were the two most important features.

**Conclusion:**

Integration of clinical parameters into a radiomics machine learning model improves prediction of the source of aldosterone overproduction and subtyping in patients with PA.

## 1 Introduction

Arterial hypertension is the leading underlying cause of cardiovascular disease and the worldwide leading modifiable risk factor for premature death (1). Its early detection, treatment and prevention of major cardiovascular events such as stroke or myocardial infarction allows to reduce both morbidity and mortality (1, 2).

Primary hyperaldosteronism (PA), also known as Conn’s syndrome, has been recognized as the most frequent cause of endocrine hypertension, characterized by an excess of aldosterone production, autonomous of major regulators of aldosterone secretion (3). PA is often underdiagnosed because of the lack of specific, easily identifiable features (4). Compared to patients with essential hypertension as well as to the general population, patients with PA have an increased risk of cerebrovascular and cardiovascular events and target organ damage (5–8).

The diagnostic workup of PA consists of a sequence of screening, confirmatory testing and differentiation of the source of aldosterone overproduction (lateralization) (9). The distinction between unilateral and bilateral primary hyperaldosteronism is crucial, because unilateral PA, most often caused by an aldosterone-producing adenoma (APA), can be cured with laparoscopic unilateral adrenalectomy, whereas bilateral PA, most often caused by bilateral hyperplasia (BHA), is routinely treated with mineralocorticoid receptor antagonists (MRA) (9).

Adrenal venous sampling (AVS) is currently considered the gold standard to distinguish unilateral from bilateral disease in patients with PA (10). AVS is technically demanding, especially because the right adrenal vein is small and may be difficult to cannulate; the success rate highly depends on the expertise of the interventional radiologist (11). AVS comes at high cost, is associated with considerable radiation exposure and is variable across different centers due to insufficient standardization of the procedure (12). Due to these challenges, AVS is not consistently available even across high-income countries.

Non-invasive imaging via computed tomography and magnetic resonance imaging (CT/MRI) has not been proven to be a reliable alternative to AVS, as micro-APA (≤10 mm in diameter) remain often undetected by those imaging methods (13–15). Another challenge in non-invasive imaging is the proportion of patients with hormonally inactive adrenal incidentalomas increasing with age, leading to an increased rate of false-positive imaging findings and reduced specificity. A meta-analysis by Zhou et al. (14) revealed that CT/MRI interpretation by radiologists has a modest pooled sensitivity and specificity in identifying unilateral PA (68% and 57%). Functional imaging techniques with C11-metomidate and imaging-based clinical scores showed inconsistent results or were not feasible on a large scale (16). Therefore, current US Endocrine Society as well as the Japan Endocrine Society guidelines recommend performance of AVS as reference standard in all patients eligible for surgical treatment (17, 18).

Quantitative image analysis offers potential solutions by introducing novel imaging biomarkers. A recent pilot study with a small patient cohort (19) demonstrated that quantitative texture analysis might allow for PA subtyping. The combination of quantitative image analysis with machine learning leads to “radiomics”, which plays an increasingly important role in the establishment of imaging biomarkers (20–23).

The aim of this study was to investigate the value of a radiomics approach supplemented by clinical and laboratory data for the prediction of the source of aldosterone overproduction and disease subtyping in patients with primary hyperaldosteronism.

## 2 Materials and methods

### 2.1 Patients

**Figure 1:**
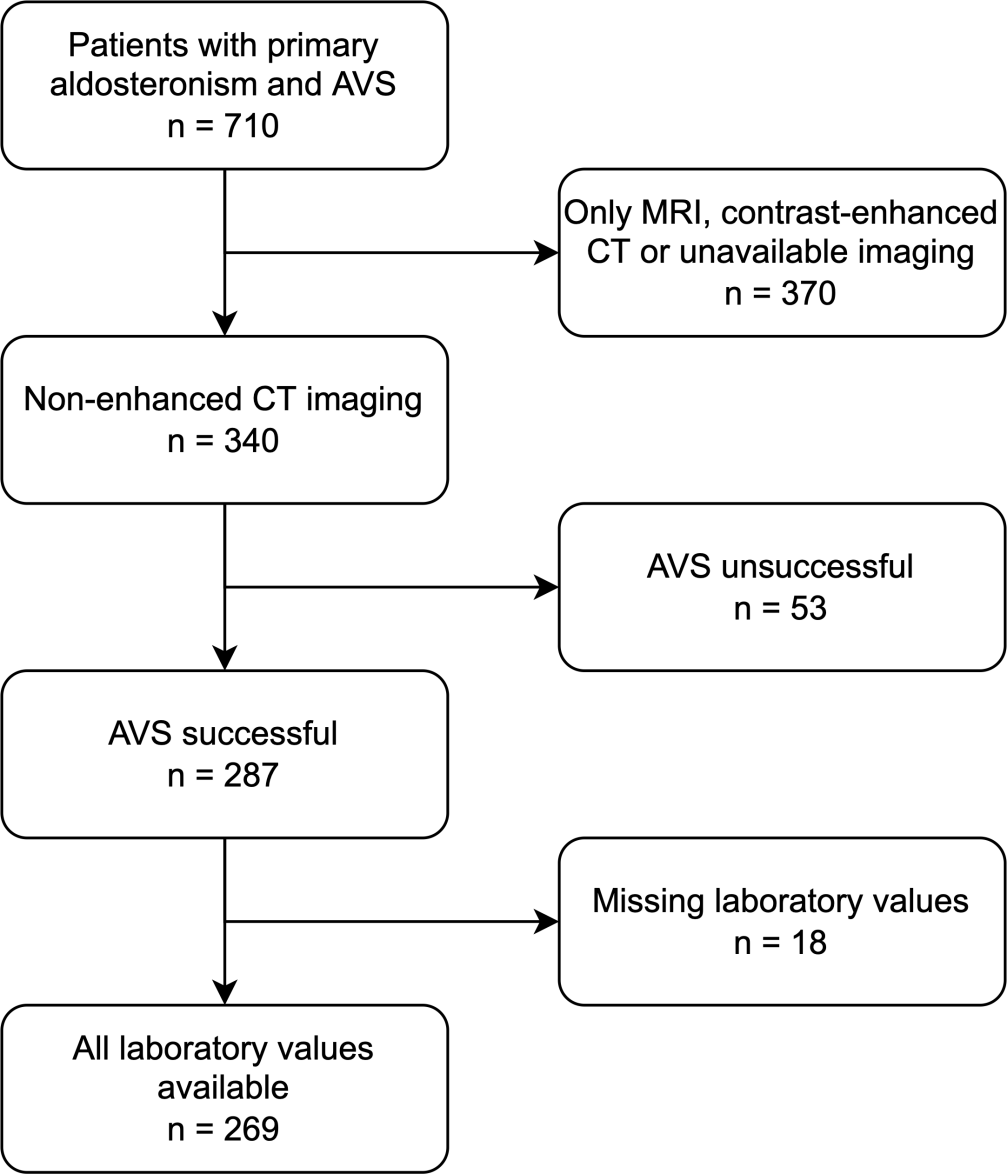
Flowchart showing the selection of the final cohort.

269 patients with confirmed primary hyperaldosteronism and CT-imaging from the German Conn Registry with diagnosed PA were enrolled (**Figure 1**). All patients underwent clinical testing, CT imaging and selective AVS performed by experienced interventional radiologists at the university hospital at LMU Munich, Germany. Inclusion criteria were successful bilateral AVS sampling and sufficient CT image quality, including pre-contrast images; exclusion criteria were equivocal AVS results and/or no suitable CT imaging or laboratory data. Patients were included prospectively in the registry; the present study was performed retrospectively as a *post-hoc* analysis. All patients gave written informed consent, and the protocol of the German Conn Registry was approved by the Ethics Committee of the medical faculty of the Ludwig Maximilians University Munich. The diagnostic procedures were performed according to the Endocrine Society Practice Guidelines (17).

### 2.2 Clinical and laboratory data

Laboratory data and blood pressure values used for analysis were acquired during the baseline visit. Blood samples were drawn in a fasting state in a sitting position in the morning. Plasma aldosterone and renin concentration were measured using the DiaSorin LIAISON® (DiaSorin Ltd, Wokingham, Berkshire, UK). Blood pressure measurements were performed in the sitting position with uncrossed legs, and the arm cuff was placed at the heart level. Blood pressure readings were obtained at the same site after not less than ten minutes of rest using a validated automatic oscillometric device. Lowest potassium concentration was defined as the lowest recorded potassium concentration in the patient’s history within two years before therapy. The establishment of the diagnosis PA took place after at least one positive confirmatory test (volume overload test or captopril test). Before testing, the antihypertensive medication was adapted according to guidelines (17). For the measurement of aldosterone post saline infusion test (SIT), 2 liters of 0.9% saline IV are given over a period of 4 hours (08:00-12:00 am). Blood samples for aldosterone, renin, cortisol, and electrolytes are drawn at time zero and after 4h. Patients remain in a seated position for at least 30 min during the infusion. If the aldosterone after saline infusion is ≥60 pg/ml, the diagnosis of PA is confirmed (with parallel suppressed renin). After confirmation further diagnosis (imaging) is required.

### 2.3 Adrenal venous sampling

In all cases, AVS was performed without adrenocorticotropic hormone stimulation in a sequential manner. During AVS, both plasma cortisol and plasma aldosterone concentration (PAC) were determined in blood collected selectively from the adrenal veins and simultaneously from the inferior vena cava (IVC). To evaluate the success of adrenal vein catheterization, selectivity index (SI) was defined as the ratio of plasma cortisol concentration for each adrenal vein and IVC (24):


$$SI\;=\;\frac{PAC\;adrenal\;veins}{PAC\;IVC}$$

If the selectivity index exceeded two, catheterization was considered successful. The lateralization index (LI) was defined as the aldosterone to cortisol ratio (ACR) on the dosssminant side with excess aldosterone secretion over ACR on the non-dominant side (25):


$$LI\;=\;\frac{ACR\;dominant\;adrenal\;vein}{ACR\;non-dominant\;adrenal\;vein}$$

If the LI exceeded four, the patient was judged to have unilateral PA. Otherwise, a diagnosis of bilateral PA was made. The results of AVS then served as the standard of truth.

### 2.4 Image analysis

For *post-hoc* analyses of CT images, we performed manual whole organ segmentation of the adrenal glands in non-contrast-enhanced CT-images (slice thickness 3-5 mm) on both sides using a dedicated segmentation software (mint lesion™, Mint Medical, Heidelberg, Germany). Non-contrast enhanced CT images were used for the purpose of unifying the study protocol as the impact of contrast enhancement in different phases on density and therefore on some of the radiomic features is not known. Adrenal glands were segmented manually by one radiologist with extensive experience in abdominal CT-imaging. To assess the inter-reader variability of the segmentations, a second reader with comparable experience annotated 50 randomly selected patients within the cohort in a second round, blinded to the results of the first reader. The agreement of segmentations of both readers were assessed via the Dice coefficient.

During pre-processing, each slice was resampled to an in-plane resolution of 1x1 mm^2^. No resampling in the z-axis was performed. We performed thresholding on pixel intensities in the range of (-300 to 300) Hounsfield Units (HU) to exclude outliers in the segmentation. A Laplacian of Gaussian filter for edge enhancement was applied to the images with σ = 1. For gray value discretization, a fixed bin size of FBS = 25 HU was used.

**Figure 2:**
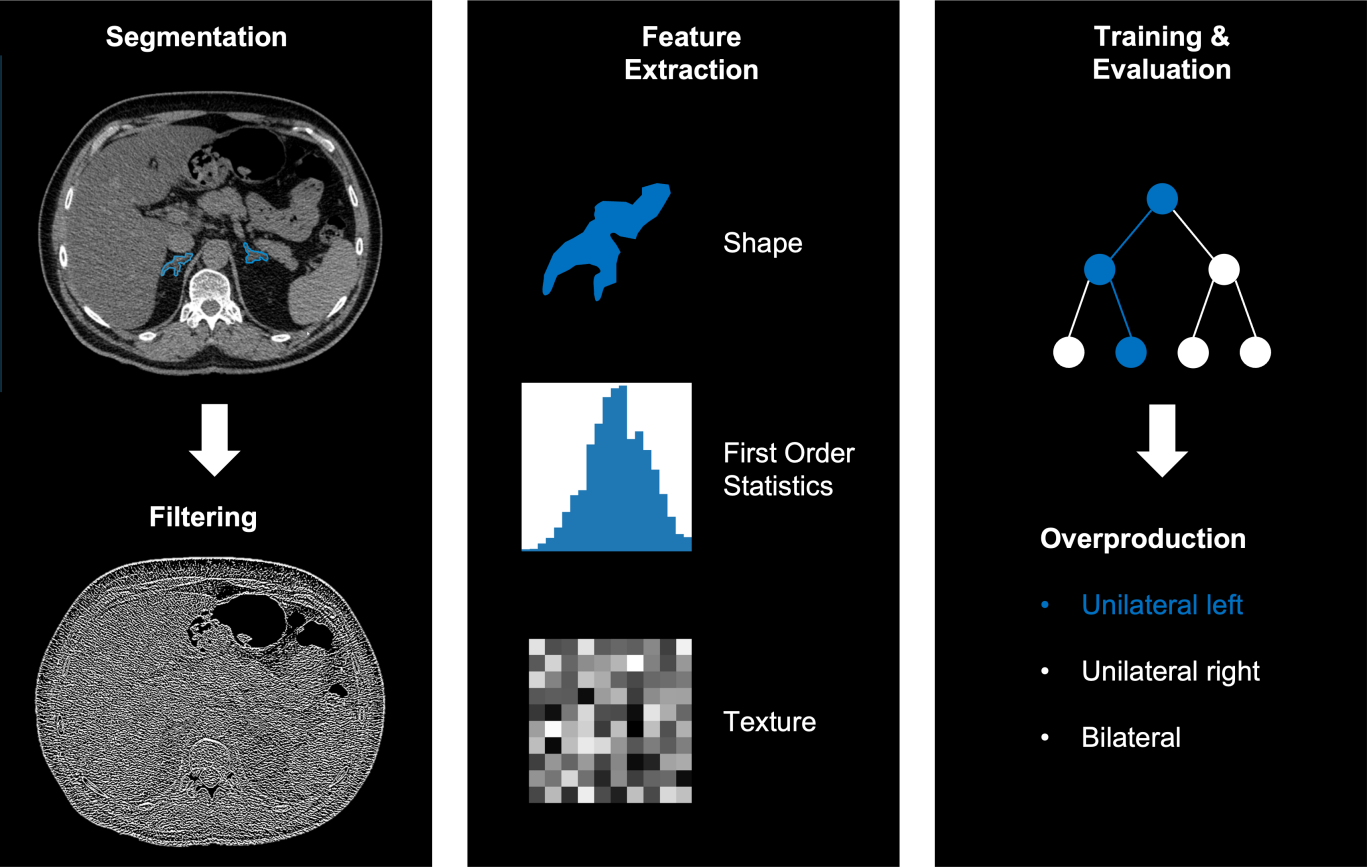
Example workflow of image segmentation and filtering (left), radiomics feature extraction (middle) and training and evaluation using a random forest (right).

We extracted 144 independent radiomic features from the segmentation of each adrenal gland using the dedicated segmentation software (mint lesion™, Mint Medical GmbH, Heidelberg, Germany), resulting in 288 features per patient (for left and right adrenal gland). The extracted features are a subset of standardized features according to the image biomarker standardization initiative (IBSI) (26). The workflow of the radiomics analysis including segmentation, filter application, feature selection and model training, and evaluation is illustrated in **Figure 2**.

### 2.5 Statistical analysis

Continuous variables were presented as mean (± standard deviation), categorical variables as the count in each subgroup. Kruskal-Wallis H-test and Chi-square test were used for global comparison of the patients’ characteristics, as appropriate, using the Python package *scipy* (version 1.9.3). Dunn’s test with Benjamini-Hochberg corrections was used for pairwise comparisons using the Python package *scikit-posthocs* (version 0.7.0).

The complete dataset was split into a stratified training and test set, according to an 80:20 split ratio. The features were standardized to zero mean and scaled to unit variance. To reduce the dimensionality, k = 25 features were selected using maximum-relevance minimum-redundancy feature selection (MRMR). These features were used to train a random forest classifier with n = 1000 estimators (implemented in Python 3.9.5 using scikit-learn 1.1.0) to predict the results of AVS, with the three target classes “unilateral left” (UL), “unilateral right” (UR), “bilateral” (BL) overproduction. A random forest classifier is an ensemble machine learning method that combines multiple decision trees to make predictions and classify data by aggregating the results of the individual trees. The predictive performance of the model was assessed with the one-versus-all area under the receiver operating characteristic curve (ROC AUC) evaluated on the hold-out test set. Confidence intervals (CI) were assessed using a bootstrap approach. Feature importance was determined for the test set using permutation feature importance which defines the decrease in model score after random shuffling of a single feature value.

For the baseline setting, we used radiomics features alone to train the model and predict the AVS result for the test set. Then, we included clinical and laboratory parameters into the model and repeated the training and evaluation process.

## 3 Results

### 3.1 Patients

**Figure 3:**
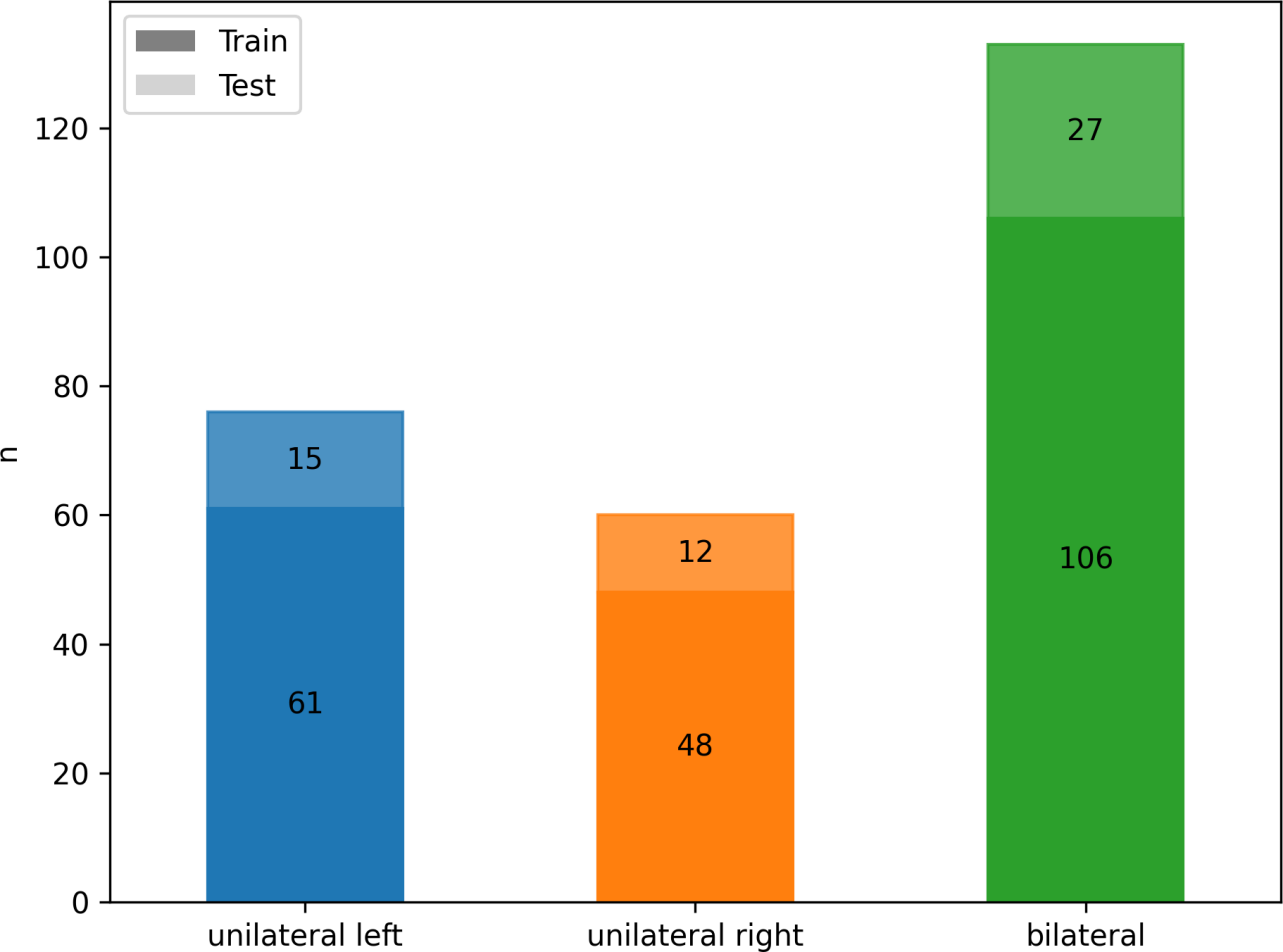
Class frequency n for the whole cohort divided by classes (color) and training (n = 215, darker) and testing (n = 54, lighter) data.

According to AVS results, 133/269 (49%) patients were diagnosed with bilateral hyperplasia, and 136 (51%) patients with unilateral aldosterone producing adenoma (n = 76 affecting the left adrenal gland, n = 60 the right). Patients with equivocal AVS results and/or no suitable CT imaging or laboratory data (n= 71) were excluded. There were no significant differences in patients’ age, sex and body mass index (BMI) between the three groups, as summarized in **Table 1**. The cohort was divided by a ratio of 80:20 into training (n = 215) and test set (n = 54) with stratified distributions of subtypes of PA, as illustrated in **Figure 3**.

**Table 1:** Patient characteristics and clinical data.

	Bilateral disease	Unilateral disease (left)*	Unilateral disease (right)*	*p*-value
Age at diagnosis [years]	51.14 (10.64)	51.58 (11.63)	52.07 (11.98)	0.703
Sex [m/f]	75/58	48/28	44/16	0.078
BMI [kg/m^2^]	28.33 (4.7)	27.88 (4.89)	27.87 (4.45)	0.722
Laboratory parameters				
Lowest level of potassium [mmol/L]	3.53 (0.46)	3.03 (0.59)^bi^	3.22 (0.5)^bi^	**< 0.001**
Sodium [mmol/L]	141.17 (2.29)	140.61 (2.97)^ur^	141.68 (2.14)	**0.031**
Total cholesterol [mg/dL]	195.9 (40.15)	191.46 (33.76)	185.13 (35.16)	0.207
HDL-C [mg/dL]	54.54 (15.14)	57.2 (15.47)	51.2 (13.36)	0.074
LDL-C [mg/dL]	121.42 (35.82)	114.7 (32.76)	111.95 (32.52)	0.206
Triglycerides [mg/dL]	120.4 (67.51)	111.61 (69.61)	124.97 (69.47)	0.18
GFR [mL/min/1,73m^2^]	82.55 (21.13)	88.06 (20.19)	83.89 (17.19)	0.16
Creatinine [mg/dL]	0.96 (0.37)	0.89 (0.23)	0.94 (0.16)	0.054
UA [mg/dL]	28.05 (11.67)	22.2 (8.88)^bi^	23.1 (9.02)^bi^	**< 0.001**
Aldosterone baseline [ng/L]	189.56 (108.07)	283.97 (275.46)^bi^	223.05 (127.78)^bi^	**< 0.001**
Aldosterone 4h after SIT [ng/L]	119.09 (70.16)	209.69 (213.39)^bi^	159.19 (117.37)^bi^	**< 0.001**
Direct Renin Concentration 4h after SIT [mU/L]	5.05 (5.65)	4.94 (4.63)	6.44 (9.56)	0.822
Mean systolic 24h-BP [mmHg]	155.2 (17.23)	151.91 (21.88)	152.08 (20.42)	0.423
Mean diastolic 24h-BP [mmHg]	96.21 (12.32)	91.8 (12.5)	94.56 (12.53)	0.059
Aldosterone-renin-ratio [ng/mU]	58.83 (46.02)	123.31 (197.75)^bi^	85.98 (106.47)	**0.049**
Aldosterone-ratio (before/after SIT)	1.74 (0.88)	1.58 (0.91)^bi^	1.73 (0.97)	**0.032**
Treatment, n (%)				
Adrenalectomy	/	53 (70%)	41 (69%)	NA
MRA	133 (100%)	17 (23%)	16 (26%)	NA
Pending adrenalectomy	/	6 (7%)	3 (5%)	NA

Data are presented as mean (± SD) unless indicated otherwise. Kruskal-Wallis H-test was used for global p-values, Dunn’s test with Benjamini-Hochberg corrections for pairwise comparisons.

24h-BP, 24-hour blood pressure; ARR, aldosterone/renin ratio; MRA, mineralocorticoid receptor antagonist; PA, primary hyperaldosteronism; HDL, high density lipoprotein cholesterol, LDL, low density lipoprotein cholesterol; GFR, glomerular filtration rate; SIT, saline infusion test; SD, standard deviation; UR, uric acid.

* A lateralization index greater than 4.0, or a lateralization index between 3 and 4 together with a contralateral index below 1.0 were considered to be compatible with unilateral disease.

^bi^ Compared to patients with bilateral disease, p< 0.05.

^ur^ Compared to patients with unilateral disease (right), p< 0.05.

Bold values indicate a p-value under < 0.05.

The interreader variability for the segmentations of 50 randomly selected patients yielded a mean Dice coefficient of 0.919, indicating a very high agreement between readers.

### 3.2 Radiomics analysis

**Figure 4:**
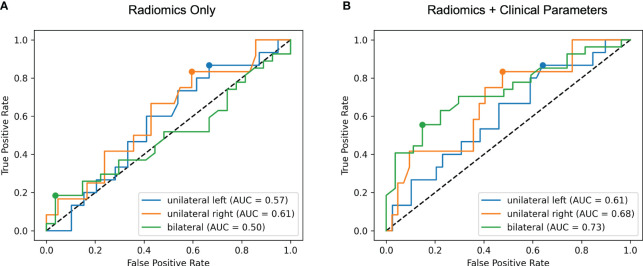
Receiver operating characteristic (ROC) curves for radiomics only **(A)** and radiomics + clinical parameters **(B)**. The area under the ROC curve (AUC) for each of the three classes unilateral left, unilateral right and bilateral is calculated in a one-versus-rest manner. Including clinical features results in higher ROC AUC values for all classes.

With the results of AVS as ground truth, the model trained with radiomics features alone discriminated the location of aldosterone overproduction with ROC AUC of 0.57 for unilateral producing adenoma affecting the left adrenal gland (UL), 0.61 affecting the right adrenal gland (UR) and 0.50 for bilateral hyperplasia (BL) (total 0.56, 95% CI: 0.45-0.65) as illustrated in **Figure 4A**. Integration of clinical and biochemical data into the machine learning model yielded better ROC AUC values regarding subtype classification for lateralization (0.61 UL, 0.68 UR, and 0.73 for BI, total 0.67, 95% CI: 0.57-0.77), displayed in **Figure 4B**.

**Figure 5:**
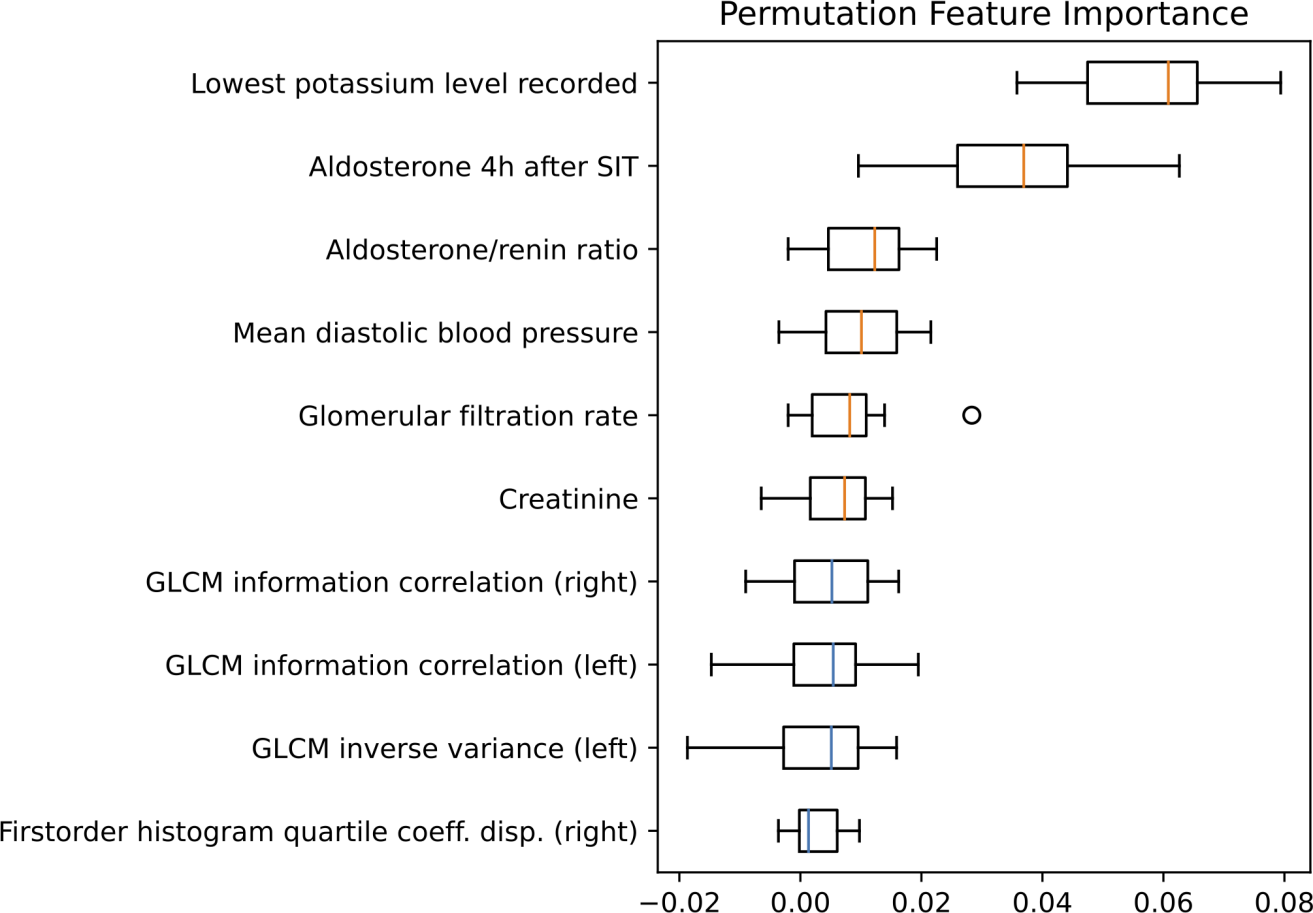
Permutation feature importance evaluated for the test set. Boxplots show the feature importance for the ten most important features. The vertical orange lines in the boxplots represent the median value of clinical features and the blue of radiomic features. GLCM, Gray level co-occurrence matrix; SIT, saline infusion test.

Using MRMR feature selection, a mix of clinical and radiomic features were selected for model training. Permutation feature importance (**Figure 5**) reveals that the most important features for the model are clinical or biochemical parameters: lowest potassium level recorded, SIT after 4 hours, aldosterone/renin ratio (ARR) and mean diastolic blood pressure measured over 24 hours are the most important distinguishing factors in subtype classification. Radiomic features, including gray level co-occurrence matrix (GLCM) features and first order features were amongst the ten most important features.

## 4 Discussion

In this explorative study, we have evaluated the value of a radiomics based approach for radiological analysis in patients with primary hyperaldosteronism. We found that the combination of quantitative image features together with clinical and laboratory parameters improves the prediction of the source of aldosterone overproduction as compared to prediction with image features alone. Our model thereby was not limited to the differentiation of aldosterone producing adenoma versus bilateral hyperplasia, but additionally aided in lateralization of the source of aldosterone overproduction. Specifically, the integration of clinical parameters (i.e. serological and clinical tests) into the prediction model led to a marked increase of ROC AUC values, with the best results achieved in the detection of BL hyperplasia. The difference in performance between UL and UR is within the statistical uncertainty, as the confidence intervals of the calculated ROC AUC values overlap due to the small sample size. Of the ten most important features, six were clinical and laboratory parameters, with lowest potassium level as the most important parameter. For the given three-class setting, this is reasonable, since spontaneous hypokalemia is a typical feature of PA, which has been shown to be present in 50% of patients with aldosterone-producing adenoma and in only 15% of patients with bilateral hyperplasia (27).

We computed radiomics features from manual segmentations of the adrenal glands. Our results indicate that these image features alone have only limited predictive value for the discrimination of PA subtypes, yet they are still represented in the top ten features for the integrated model. While the predictive value of image features might be improved, e.g. through optimized and standardized image acquisition or automated adrenal gland segmentation, our findings highlight the value of an integrated diagnostics approach. This observation is in line with a recent study which demonstrated that discrimination of tumor recurrence from radiation necrosis in glioma patients is improved with an integrated model approach (28).

Several prediction models have been investigated in the past to improve the diagnostics of PA. Models analyzing clinical and laboratory data have been proven to be successful in predicting the subtype of PA. Shi et al. (29) investigated 10 clinical/laboratory features and found the primary aldosterone concentration after SIT, aldosterone-to-renin-ratio and ARR after captopril challenge test, as the most important variables contributing to the model. Tamaru and colleagues (30) proposed a similar model to predict PA subtype (bilateral vs. unilateral). Both models thus successfully aided in subtype differentiation – localized adenoma versus hyperplasia - however without imaging information lateralization of a unilateral aldosterone producing adenoma is not possible.

We found that the top ten overall features for the integrated model comprise clinical, laboratory and imaging features. In particular, potassium level and aldosterone-to-renin ratio are relevant for PA subtyping (i.e. hyperplasia yes or no), whereas imaging features are relevant for prediction of disease lateralization. These results agree with findings from a recent study (31), which aimed to predict which unilateral adenomas over-produce aldosterone. Similar to our findings, age, sex, serum potassium level and aldosterone-to-renin ratio remained as independent predictors after variable selection. Furthermore, aldosterone concentration after a four-hour SIT contributed substantially to an improvement of the prediction model. This is in line with Nagano et al. (32) who found plasma aldosterone concentrations below 87.9 pg/ml after a SIT as discriminatory predictor for bilateral primary aldosteronism. When comparing several confirmatory tests, a recent meta-analysis showed that the SIT had the best sensitivity and specificity for predicting the subtype of primary aldosteronism (33). In another integrated prediction model, aldosterone levels at screening and after confirmatory testing, as well as lowest potassium level were shown to be the most important clinical parameters predicting subtype diagnosis in primary aldosteronism (34). However, dichotomization into lateralized and bilateral disease in this prediction model does not represent the complexity of the disease as it fails to define with certainty the side of aldosterone hypersecretion, which is highly relevant in lateralized cases eligible for surgical treatment. Furthermore, the presence of visible nodules in CT-imaging in approximately 86% of the lateralized cases possibly caused a preselection bias.

The SPARTACUS trial (35) compared the outcome of CT-based diagnosis combined with AVS-based management in patients with PA who were treated with either adrenalectomy or MRA and were followed one year. The study did not find significant differences in intensity of antihypertensive medication or clinical benefits. Due to multiple limitations of the study, however, the only randomized prospective trial in this field could not refute the view that AVS is the gold standard for subtyping PA. In light of the SPARTACUS trial, conducting treatment decisions in primary aldosteronism on AVS results alone is challenged without offering a suitable alternative. With a nearly 50% discordance between the diagnostic conclusions derived from the CT and AVS (35), the portion of patients with aldosterone-producing micronodules treated permanently with MRA instead of surgical treatment remains unknown. Recent data by Williams et al. (36) also displayed beneficial outcomes of adrenal surgery for selected patients with bilateral primary aldosteronism, challenging the current dogma of routine medical management of this disease subtype.

Our data indicate that aldosterone overproduction caused by micronodules still may not be detectable by a quantitative analysis of medical imaging like radiomics in a substantial number of patients. This is consistent with findings at our center that reported a prevalence of nonclassical histopathology of unilateral PA in 25% of the unilateral cases, which were mainly attributed to multiple aldosterone-producing micronodules (37).

This study is not without limitations. As a consequence of non-standardized imaging protocols, imaging data is heterogeneous and introduces noise in quantitative image features. Further studies would benefit from standardized CT imaging or even MR imaging with a superior soft-tissue contrast. Moreover, functional imaging techniques, such as Positron Emission Tomography (PET), potentially hybridized with CT or MRI would allow more advanced contrast options with the possibility of molecular imaging (38). Combing those advanced imaging technologies with functional testing such as provocative or inhibitory test may allow for non-invasive diagnostics of PA in the future. Manual segmentation of adrenal glands is tedious; Although we observed very good inter-reader agreement, reproducibility might be further increased through automated approaches, e.g. with U-net based deep learning approaches. We did not perform hyperparameter tuning or benchmarking for our prediction model, simply because the sample size did not allow for the necessary additional test dataset. However, we used a random forest model with robust default settings which has proven to be suitable for classification tasks with a large number of features (39). Our reference standard for PA subtype differentiation in this study was AVS. Although AVS is the current reference standard recommended by experts and guidelines for subtype diagnosis in PA (17), its role in the management of patients PA is currently under debate (35). Additionally, a subgroup of patients affected by non-secreting adrenal incidentalomas of various size would have been an interesting comparison, however within the German Conn registry this group was not available.

## 5 Conclusion

This study revealed that an integrated diagnostics approach can notably improve the non-invasive identification of the source of aldosterone overproduction and subtype differentiation in PA, when compared to a radiomics approach using CT-imaging data alone. Before such an integrated approach can replace invasive AVS, the predictive value must be further improved and needs to be validated in prospective interventional studies, where machine learning approaches guide clinical decision making.

## Data availability statement

The raw data supporting the conclusions of this article will be made available by the authors, without undue reservation.

## Ethics statement

All procedures performed in studies involving human participants were in accordance with the ethical standards of the institutional research committee and with the 1964 Helsinki declaration and its later amendments or comparable ethical standards. Data collection was according to the protocol of the German Conn’s Registry approved by the Ethics Committee of the Medical Faculty of the Ludwig Maximilians University of Munich. All patients gave written informed consent, and the protocol of the German Conn’s Registry was approved by the Ethics Committee of the University of Munich.

## Author contributions

NM, AM, RW, MW, and MI: conception and design of the study, generation, collection, assembly, analysis, and/or interpretation of data, drafting or revision of the manuscript, and approval of the final version of the manuscript. BS, JaR, DH, DB, LT, HN, MB, SK, CA, JeR, MR, and NR: generation, collection, assembly, analysis, and/or interpretation of data; drafting or revision of the manuscript; approval of the final version of the manuscript.
